# Optimization of the Capsid of Recombinant Adeno-Associated Virus 2 (AAV2) Vectors: The Final Threshold?

**DOI:** 10.1371/journal.pone.0059142

**Published:** 2013-03-19

**Authors:** George V. Aslanidi, Angela E. Rivers, Luis Ortiz, Liujiang Song, Chen Ling, Lakshmanan Govindasamy, Kim Van Vliet, Mengqun Tan, Mavis Agbandje-McKenna, Arun Srivastava

**Affiliations:** 1 Division of Cellular and Molecular Therapy, Department of Pediatrics, University of Florida College of Medicine, Gainesville, Florida, United States of America; 2 Powell Gene Therapy Center, University of Florida College of Medicine, Gainesville, Florida, United States of America; 3 Genetics Institute, University of Florida College of Medicine, Gainesville, Florida, United States of America; 4 Division of Hematology/Oncology, Department of Pediatrics, University of Illinois at Chicago, Chicago, Illinois, United States of America; 5 Experimental Hematology Laboratory, Department of Physiology, Xiangya School of Medicine, Central South University, Changsha, China; 6 Shenzhen Institute of Xiangya Biomedicine, Shenzhen, China; 7 Department of Biochemistry and Molecular Biology, University of Florida College of Medicine, Gainesville, Florida, United States of America; 8 Department of Molecular Genetics and Microbiology, University of Florida College of Medicine, Gainesville, Florida, United States of America; 9 Shands Cancer Center, University of Florida College of Medicine, Gainesville, Florida, United States of America; National Institute of Dental and Craniofacial Research, United States of America

## Abstract

The ubiquitin-proteasome pathway plays a critical role in the intracellular trafficking of AAV2 vectors, and phosphorylation of certain surface-exposed amino acid residues on the capsid provides the primary signal for ubiquitination. Removal of several critical tyrosine (Y) and serine (S) residues on the AAV2 capsid has been shown to significantly increase transduction efficiency compared with the wild-type (WT) vectors. In the present study, site-directed mutagenesis of each of the 17 surface-exposed threonine (T) residues was conducted, and the transduction efficiency of four of these mutants, T455V, T491V, T550V, and T659V, was observed to increase up to 4-fold in human HEK293 cells *in vitro*. The most critical Y, S, and T mutations were subsequently combined, and the quadruple-mutant (Y444+500+730F+T491V) AAV2 vector was identified as the most efficient. This vector increased the transduction efficiency ∼24-fold over the WT AAV2 vector, and ∼2–3-fold over the previously described triple-mutant (Y444+500+730F) vector in a murine hepatocyte cell line, H2.35, *in vitro*. Similar results were obtained in murine hepatocytes *in vivo* following tail vein injection of the Y444+500+730F+T491V scAAV2 vector, and whole-body bioluminescence imaging of C57BL/6 mice. The increase in the transduction efficiency of the Y-T quadruple-mutant over that of the Y triple-mutant correlated with an improved nuclear translocation of the vectors, which exceeded 90%. These observations suggest that further optimization of the AAV2 capsid by targeting amino acid residues involved in phosphorylation may not be possible. This study has thus led to the generation of a novel Y444+500+730F+T491V quadruple-mutant AAV2 vector with potential for use in liver-directed human gene therapy.

## Introduction

Adeno-associated virus (AAV) vectors are currently in use in a number of Phase I/II clinical trials as delivery vehicles to target a variety of tissues to achieve sustained expression of therapeutic genes [1], [2], [3], [4], [5]. However, large vector doses are needed to achieve therapeutic benefits. The requirements for sufficient amounts of the vector pose a production challenge, as well as the risk of initiating the host immune response to the vector [6], [7], [8]. More specifically, recombinant vectors based on AAV2 serotype were initially used in a clinical trial for the potential gene therapy of hemophilia B, but in this trial, therapeutic level of expression of human Factor IX (hF.IX) was not achieved at lower vector doses, and at higher vector doses, the therapeutic level of expression of hF.IX was short-lived due to a cytotoxic T cell (CTL) response against AAV2 capsids [9], [10], [11]. In a more recent trial with recombinant vectors based on AAV8 serotype, therapeutic levels of expression of hF.IX were been achieved, but an immune response to AAV8 capsid proteins was observed [12]. Thus, it is critical to develop novel AAV vectors with high transduction efficiency that can be used at lower doses. We have previously reported that cellular epidermal growth factor receptor protein tyrosine kinase (EGFR-PTK) negatively impacts transgene expression from recombinant AAV2 vectors primarily due to phosphorylation of AAV2 capsids at tyrosine residues, and tyrosine-phosphorylated capsids are subsequently degraded by the host proteasome machinery [13], [14]. In our more recent studies [12], we observed that selective inhibitors of JNK and p38 MAPK serine/threonine kinases also improve the transduction efficiency of AAV2 vectors, suggesting that phosphorylation of certain surface-exposed serine and/or threonine residues might also decrease the transduction efficiency of these vectors. These studies led to the development of tyrosine- and serine-mutant AAV2 vectors, which we subsequently documented to transduce various cell types with significantly higher efficiency than the WT vectors [12], [13], [14], [15]. We hypothesized that in addition to the tyrosine and serine residues, the elimination of surface-exposed threonine residues by site-directed mutagenesis, might also lead to an increase in the transduction efficiency at lower vector doses. Each of the 17 surface-exposed threonine residues was substituted with valine (V) residues by site-directed mutagenesis, and four of these mutants, T455V, T491V, T550V, T659V, were shown to increase the transduction efficiency between ∼2–4-fold in human HEK293 cells. Since we have previously reported that the tyrosine triple-mutant (Y730F+500+444F) vector transduces murine hepatocytes most efficiently than WT [12], [13], [14], [15], we subsequently combined these mutations with the best-performing single serine-mutant (S662V) and single threonine-mutant (T491V) to generate the following vectors: two quadruple (Y444+500+730F+S662V; Y730+500+44F+T491V) and one quintuple (Y444+500+730F+S662V+T491V); and tested our hypothesis of whether further improvement in transduction efficiency of these multiple-mutants could be achieved. We report here the identification of the quadruple-mutant (Y444+500+730F+T491V) vector that efficiently transduces a murine hepatocyte cell line *in vitro* as well as primary murine hepatocytes *in vivo* at reduced doses, which has implications in the potential use of these vectors in human gene therapy in general, and hemophilia in particular.

## Materials and Methods

### Cells

Human embryonic kidney cell line, HEK293, and murine hepatocyte cell line, H2.35, cells were obtained from the American Type Culture Collection (Manassas, VA), and maintained as monolayer cultures in DMEM (Invitrogen) supplemented with 10% fetal bovine serum (FBS; Sigma) and antibiotics (Lonza).

### Production of Recombinant Vectors

Recombinant AAV2 vectors containing either EGFP (scAAV2-GFP) or firefly luciferase gene (Fluc) (ssAAV2-Fluc) driven by the chicken β-actin promoter (CBA) were generated as described previously [12], [16], [17], [18]. Briefly, HEK293 cells were transfected using Polyethylenimine (PEI, linear, MW 25,000, Polysciences, Inc.). Seventy-two hrs post-transfection, cells were harvested and vectors were purified by iodixanol (Sigma) gradient centrifugation and ion exchange column chromatography (HiTrap Sp Hp 5 ml, GE Healthcare). Virus was then concentrated and buffer exchanged into Lactated Ringer’s solution in three cycles using centrifugal spin concentrators (Apollo, 150-kDa cut-off, 20-ml capacity, CLP). To determine genome titers, ten µl of purified virus were incubated with DNase I (Invitrogen) at 37°C for 2 h, then with Proteinase K (Invitrogen) at 55°C for an additional 2 h. The reaction mixture was purified by phenol/chloroform, followed by chloroform extraction. Packaged DNA was precipitated O/N with ethanol in the presence of 20 µl glycogen (Invitrogen). DNase I-resistant AAV2 particle titers were determined by qPCR with the following primer-pairs specific for the CBA promoter: F-5′-TCCCATAGTAACGCCAATAGG-3′, R-5′-CTTGGCATATGATACACTTGATG-3′ and SYBR GreenER PCR Master Mix (Invitrogen) [12], [16].

### Site-directed Mutagenesis

A two-stage PCR was performed with plasmid pACG2 as described previously [12], [19] using Turbo *Pfu* Polymerase (Stratagene). Briefly, in stage one, two PCR extension reactions were performed in separate tubes for the forward and reverse PCR primers for 3 cycles. In stage two, the two reactions were mixed and a PCR reaction was performed for an additional 15 cycles, followed by Dpn I digestion for 1 hr. Primers were designed to introduce changes from threonine (A*CA*) to valine (*GTA*) for each of the residues mutated.

### Recombinant AAV Vector Transduction Assays *in vitro*

Human HEK293 were transduced with 1×10^3^ vgs/cell, and murine hepatocytes H2.35 cells were transduced with 2×10^3^ vgs/cell with WT and mutant scAAV2-GFP vectors, respectively, and incubated for 48 h. Transgene expression was assessed as the total area of green fluorescence (pixel^2^) per visual field (mean ± SD) as described previously [12], [13], [14]. Analysis of variance was used to compare test results and the control, which were determined to be statistically significant.

### Analysis of Vector Genome Distribution in Cytoplasm and Nuclear Fractions

Approximately 1×10^6^ H2.35 cells were infected by either WT or mutant scAAV2-GFP vectors with MOI 1×10^4^ vgs/cell. Cells were collected at various time points by trypsin treatment to remove any adsorbed and un-adsorbed viral particles and then washed extensively with PBS. Nuclear and cytoplasmic fractions were separated with Nuclear and Cytoplasmic Extraction Reagents kit (Thermo Scientific) according to manufacturer instruction. Viral genome was extracted and detected by qPCR analysis with the CBA specific primers described above. The difference in amount of viral genome between cytoplasmic and nuclear fractions was determined by the following rule: C_T_ values for each sample from cells treated with virus were normalized to corresponding C_T_ from mock treated cells (ΔC_T_). For each pairwise set of samples, fold change in packaged genome presence was calculated as fold change = 2**^−(^** ^ΔCT-cytoplasm−ΔCT-nucleus**)**.^ Data from three independent experiments were presented as a percentage of the total amount of packaged genome in the nuclear and cytoplasmic fractions.

### *In vivo* Bioluminescence Imaging

All animal experiments were approved by the University of Florida Institutional Animal Care and Use Committee. All procedures were done in accordance with the principles of the National Research Council's Guide for the Care and Use of Laboratory Animals. All efforts were made to minimize suffering. Ten-week-old C57BL/6 male mice (Jackson Laboratory, Bar Harbor, ME) were injected intravenously with 1×10^10^ vgs/animal of WT and mutant ssAAV2-Fluc vectors (n = 3). Luciferase activity was analyzed two weeks post injection using a Xenogen IVIS Lumina System (Caliper Life Sciences). Briefly, mice were anesthetized with 2% isofluorane and injected intraperitoneally with luciferin substrate (Beetle luciferin, Caliper Life Sciences) at a dose of 150 µg/g of body weight. Mice were placed in a light-tight chamber and images were collected at 5 minutes after the substrate injection. Images were analyzed by the Living Image 3.2 software (Caliper Life Sciences) to determine relative signal intensity.

### Visualization of the Position of the Mutant Residues on the AAV2 Capsid

The atomic coordinates for the AAV2 VP3 crystal structure (residues 217 to 735, VP1 numbering) (Protein Data Bank (PDB) accession no. 1lp3; [20]) was downloaded and used to generate a complete capsid model using the Oligomer generator application in VIPERdb [21]. This generates 60 VP3 copies for creating the T = 1 icosahedral capsid via matrix multiplication. The structure was viewed with the program COOT [22] and Figures were generated using either the software PyMOL (Schrodinger, LLC) or RIVEM [23].

### Statistical Analysis

Results are presented as mean ± S.D. Differences between groups were identified using a grouped-unpaired two-tailed distribution of Student’s T-test. P-values <0.05 were considered statistically significant.

## Results

### Site-directed Mutagenesis of Surface-exposed Threonine Residues on AAV2 Capsid Improves Vector-mediated Transgene Expression in Human Cells *in vitro*

**Figure 1:**
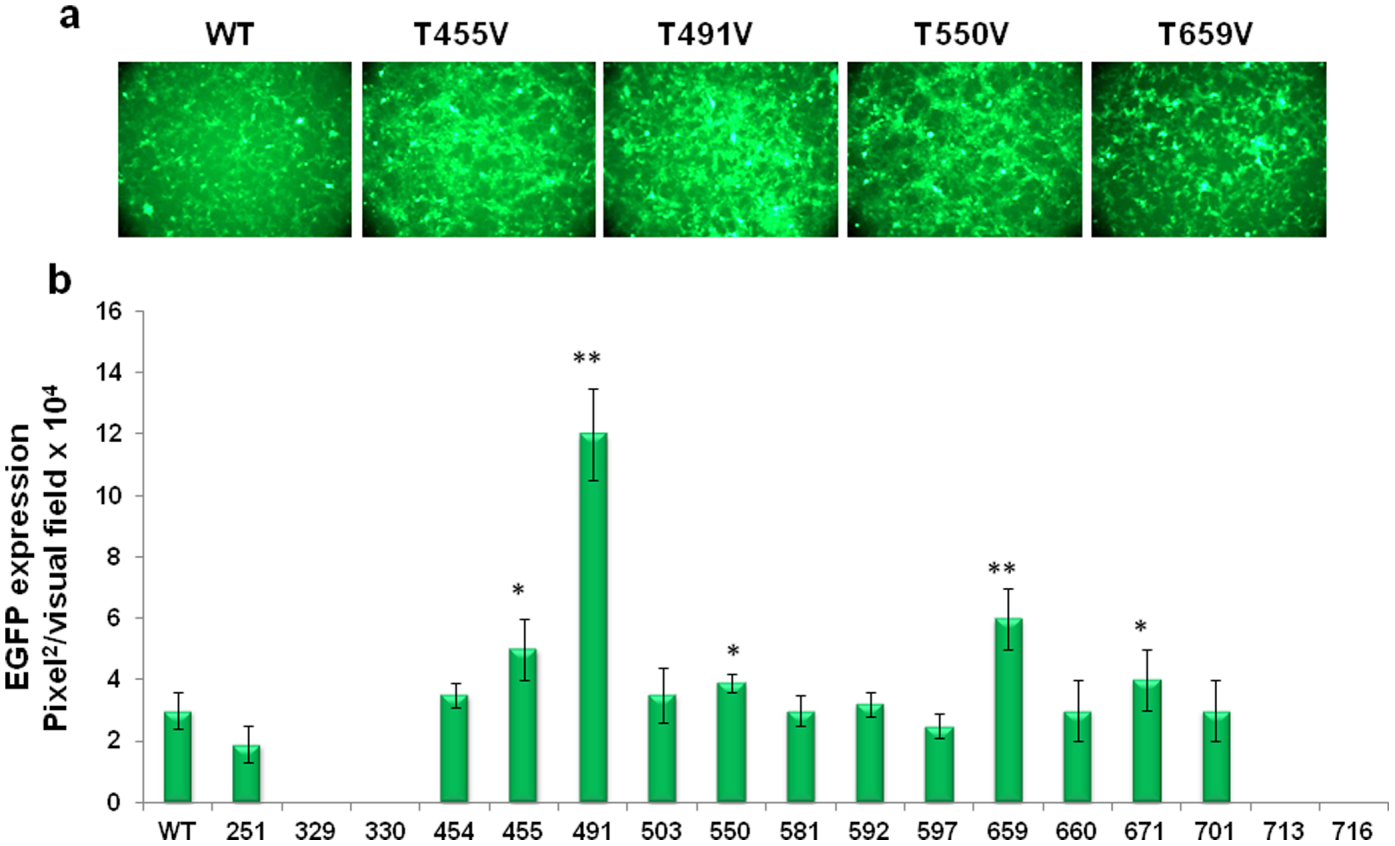
Analysis of EGFP expression after transduction of HEK293 cells with individual site-directed AAV2 capsid mutants. Each of the 17 surface-exposed threonine (T) residues in AAV2 capsid was substituted with valine (V) and evaluated for its efficiency to mediate transgene expression. (**a**) EGFP expression analysis at 48 h post-infection at MOI of 1×10^3^ vg/cell. (**b**) Quantification of transduction efficiency of each of the threonine-mutant scAAV2 vectors. *P<0.005, **P<0.001 vs. WT AAV2.

The AAV2 capsid contains 45 threonine (T) residues in the capsid viral protein 3 (VP3) common region of the three capsid VPs, VP1, VP2, and VP3. Seventeen of these (251, 329, 330, 454, 455, 491, 503, 550, 581, 592, 597, 671, 659, 660, 701, 713, 716) are surface-exposed [20]. Each of the 17 T residues was substituted with valine (V) by site-directed mutagenesis as described previously [12], [13]. Most mutants could be generated at titers similar to the WT AAV2 vectors, with the exception of T329V and T330V which were produced at ∼10-fold lower titers, and T713V and T716V, which produced no detectable levels of DNase Ι-resistant vector particles. Each of the T-V mutant vectors was evaluated for transduction efficiency in HEK293 cells. These results, shown in **Fig. 1a and b** **,** indicate that of the 17 mutants, the T491V mutant transduced HEK293 cells ∼4-fold more efficiently than its WT counterpart. The transduction efficiency of the T455V, T550V, T659V mutant vectors were increased by ∼2-fold. These data support our hypothesis that phosphorylation of specific tyrosine, serine, and threonine residues on AAV2 capsid by cellular kinases is a critical determinant of the transduction efficiency of these vectors.

### Multiple Mutations of Surface-exposed Threonine Residues Further Improve the Transduction Efficiency of AAV2 Vectors

**Figure 2:**
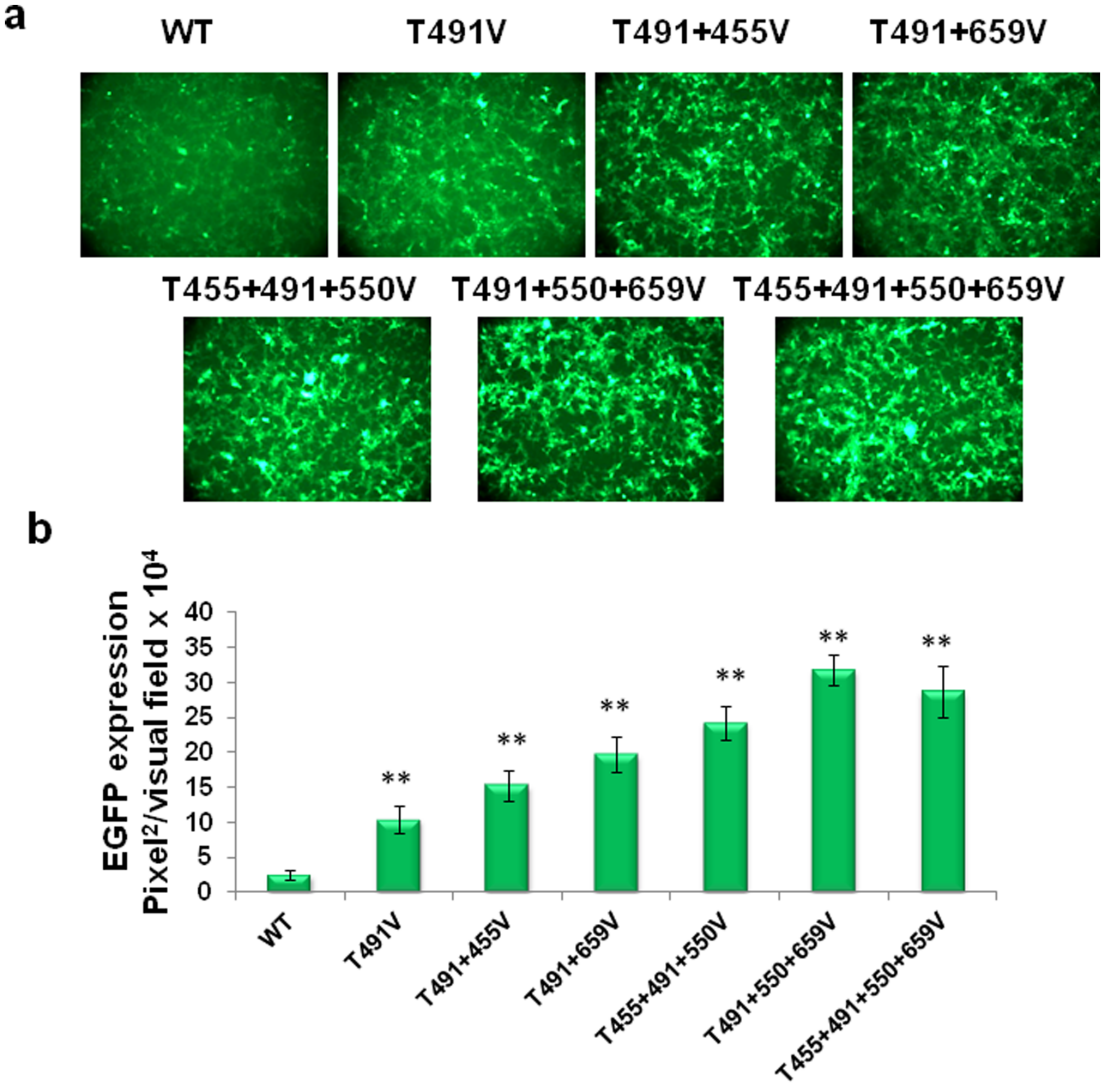
Analysis of EGFP expression in HEK293 cells infected with multiple site-directed AAV2 capsid mutants. Several most efficient threonine mutations were combined on single AAV2 capsid to produce double- and triple-mutant and efficiency of each vector was evaluated. (**a**) EGFP expression analysis at 48 h post-infection at MOI of 1×10^3^ vg/cell. (**b**) Quantification of transduction efficiency of each of the threonine-mutant AAV2 vectors. *P<0.005, **P<0.001 vs. WT AAV2.

To evaluate whether the transduction efficiency of the threonine-mutant AAV2 vectors could be enhanced further, the following multiple-mutant vectors were generated: three double-mutants (T455+491V; T550+491V; T659+491V), two triple-mutants (T455+491+550V; T491+550+659V), and one quadruple-mutant (T455+491+550+659V). Each of the multiple-mutant vectors packaged genome titers similar to the WT AAV2 vectors. In side-by-side comparisons, each of the multiple-mutant vectors was shown to transduce HEK293 more efficiently than the WT and the single-threonine mutant AAV2 vectors (**Fig. 2a,b**). The best performing vector was identified to be the triple-mutant (T491+550+659V), with the transduction efficiency ∼10-fold higher than the WT vector, and ∼3-fold higher than the best single-mutant (T491V) vector. These data suggest, as observed previously with multiple surface tyrosine-mutants [14], that combining several threonine-mutations on a single viral capsid can also lead to a synergetic effect in augmenting the transduction efficiency.

### Optimized Threonine-mutant AAV2 Vectors Efficiently Transduce Murine Hepatocytes *in vitro*

**Figure 3:**
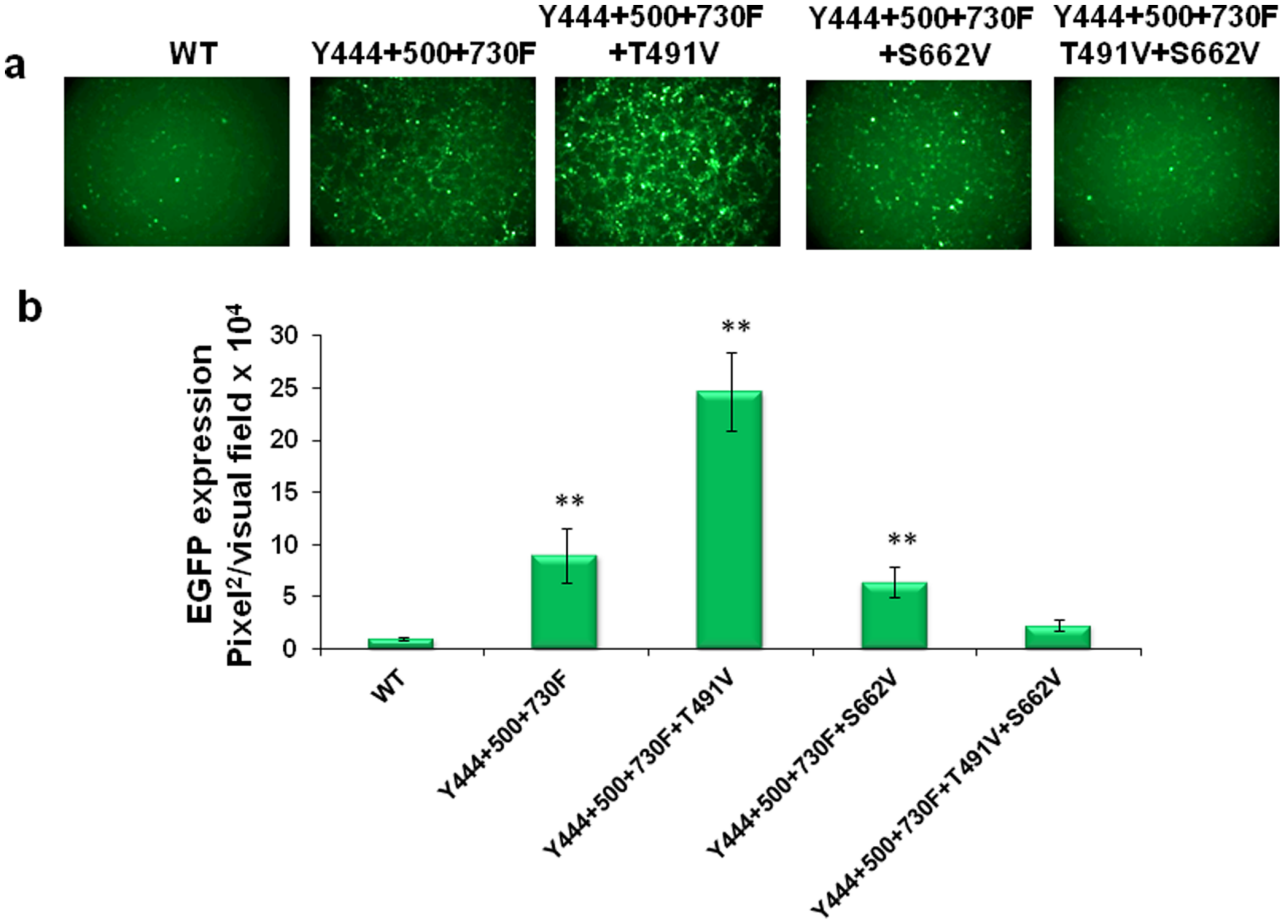
Evaluation of EGFP expression in H2.35 cell transduced with capsid optimized AAV2 vectors. The most efficient tyrosine, serine and threonine mutations were combined on single AAV2 capsid to produce several optimized AAV mutants. Efficiency of each vector was estimated on immortalized murine hepatocytes. (**a**) EGFP expression analysis at 48 h post-infection at MOI of 1×10^3^ vg/cell. (**b**) Quantification of transduction efficiency of each of the optimized scAAV2 vectors. *P<0.005, **P<0.001 vs. WT AAV2.

As stated above, we have previously reported that a tyrosine triple-mutant (Y444+550+730F) vector was the most efficient in transducing murine hepatocytes in a comparison of vectors containing up to 7 surface tyrosine to phenylalanine changes [14], [24]. Thus it was of interest to evaluate whether combining the best performing single-serine (S662V) and single-threonine (T491V) mutations with the triple-tyrosine mutant could further increase the transduction efficiency of these vectors. We generated several multiple-mutants as follows: two quadruple (Y444+500+730F+T491V; Y444+500+730F+S662V), and one quintuple (Y444+500+730F+T491V+S662V) mutant vectors. Comparison of the transduction efficiency of these mutants with the WT and the tyrosine triple-mutant AAV2 vectors in H2.35 cells showed that the expression level from the Y444+500+730F+T491V mutant was ∼2–3-fold higher than for the tyrosine triple-mutant AAV2 vector, and ∼24-fold higher than the WT AAV2 vector (**Fig. 3a,b**). Interestingly, combining the S662V mutation with the tyrosine triple-mutant vector, or with the tyrosine-threonine quadruple-mutant vector, negatively affected their transduction efficiency. Addition of several other threonine mutations, such as T550V and T659V, also did not augment the transduction efficiency of the Y444+500+730F+T491V quadruple-mutant AAV2 vector (data not shown). Additional studies are warranted to gain a better understanding of the complex interactions among these surface-exposed Y, S, and T residues as well as their phosphorylation status.

### Multiple-mutations Enhance Intracellular Trafficking and Nuclear Translocation of AAV2 Vectors

**Figure 4:**
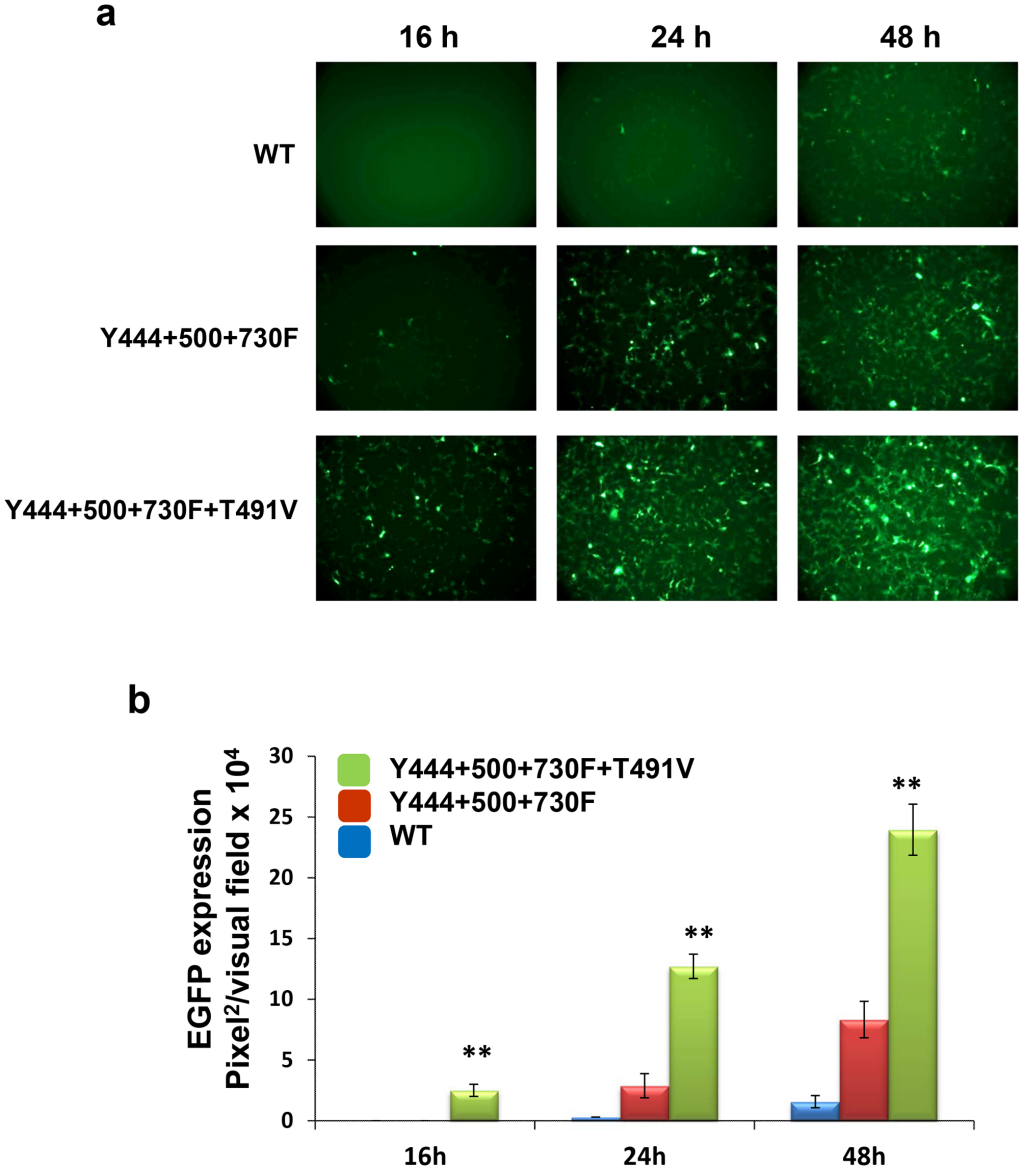
Kinetics of EGFP expression in H2.35 cell mediated by capsid optimized AAV vectors. (**a**) EGFP expression analysis at 16, 24 and 48 h post-infection at MOI of 1×10^3^ vgs/cell. (**b**) Quantification of transduction efficiency of each of the optimized scAAV2 vectors. *P<0.005, **P<0.001 vs. WT AAV2.

**Figure 5:**
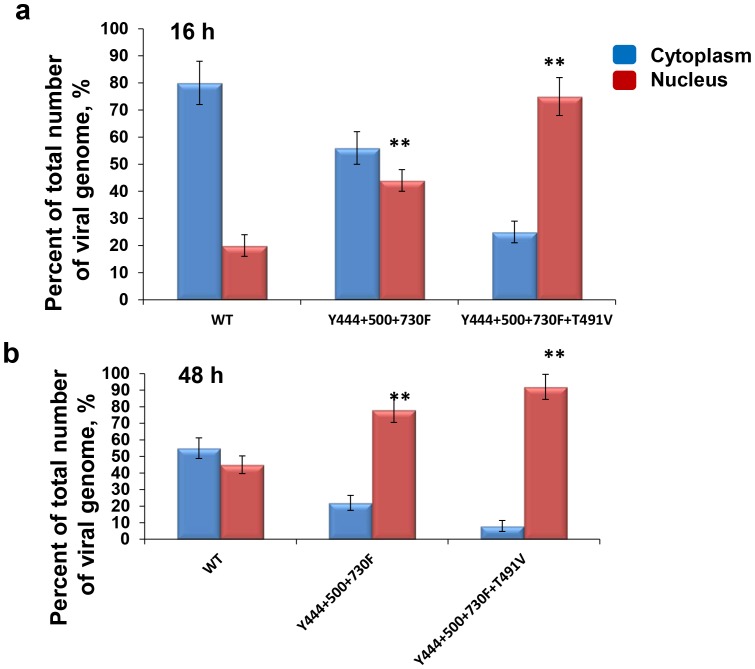
Analysis of intracellular trafficking of AAV multiple mutant vectors to the nucleus. Nuclear and cytoplasmic fraction of H2.35 cell infected with AAV2-WT, AAV2-Y444+500+730F and AAV2- Y444+500+730F+T491V mutant were separated and qPCR analysis was performed to evaluate vector genome distribution within cell in 16 h (**a**) and 48 h (**b**) post infection. **P<0.001 vs. WT in nucleus was considered as significant.

We have previously reported that prevention of phosphorylation of surface-exposed tyrosine residues on the AAV2 capsid improves intracellular trafficking of tyrosine-mutant vectors and increases the number of the viral genomes translocated to the nucleus [13], [25]. In the present studies, we wished to examine whether the addition of the T491V mutant to the tyrosine triple-mutant vector augmented the transduction efficiency by further increasing nuclear transport of these vectors. To this end, we first evaluated the kinetics of transgene expression in H2.35 cells mediated by the Y444+500+730F+T491V quadruple-mutant and compared it with the Y444+500+730F triple-mutant and the WT AAV2 vectors. These results are shown in **Fig. 4a and b**. As can be seen, EGFP expression from the tyrosine-threonine quadruple-mutant vector was ∼2–3fold higher at each tested time point, and could be detected as early as 16 h post-infection. These results suggested that the early-onset of transgene expression from the quadruple-mutant vectors could be due to more efficient nuclear transport of these vectors. To experimentally test this possibility, we next used qPCR analysis to quantitate the vector genomes in cytoplasmic and nuclear fractions of H2.35 cells infected with the WT and the two mutant AAV2 vectors at different time points. The vector genome ratios in the two cellular fractions are shown in **Fig. 5a,b**. Consistent with previously published data [13], [25], [26], [27], [28], [29], whereas ∼20% of the genomes from the WT AAV2 vectors, and ∼45% of the genomes from the triple-mutant vectors were detected in the nuclear fraction 16 h post-infection, more than 70% of the vector genomes from the quadruple-mutant were detected at the same time-point. Similarly, only ∼45% of the genomes from the WT AAV2 vectors were detected in the nuclear fraction 48 hrs post-infection, ∼80% of the genomes from the triple-mutant vectors, and ∼90% of the vector genomes from the quadruple-mutant were detected in the nuclear fraction at the same time-point. Thus, these data corroborated our hypothesis that combining the threonine (T491V) mutation with the tyrosine triple-mutant (Y444+500+730F) vector leads to a modest improvement in the nuclear translocation of these vectors, which correlated with a faster onset of gene expression and the observed improvement in the transduction efficiency.

### Optimized AAV2 Vectors are Highly Efficient in Transducing Murine Hepatocytes *in vivo*

**Figure 6:**
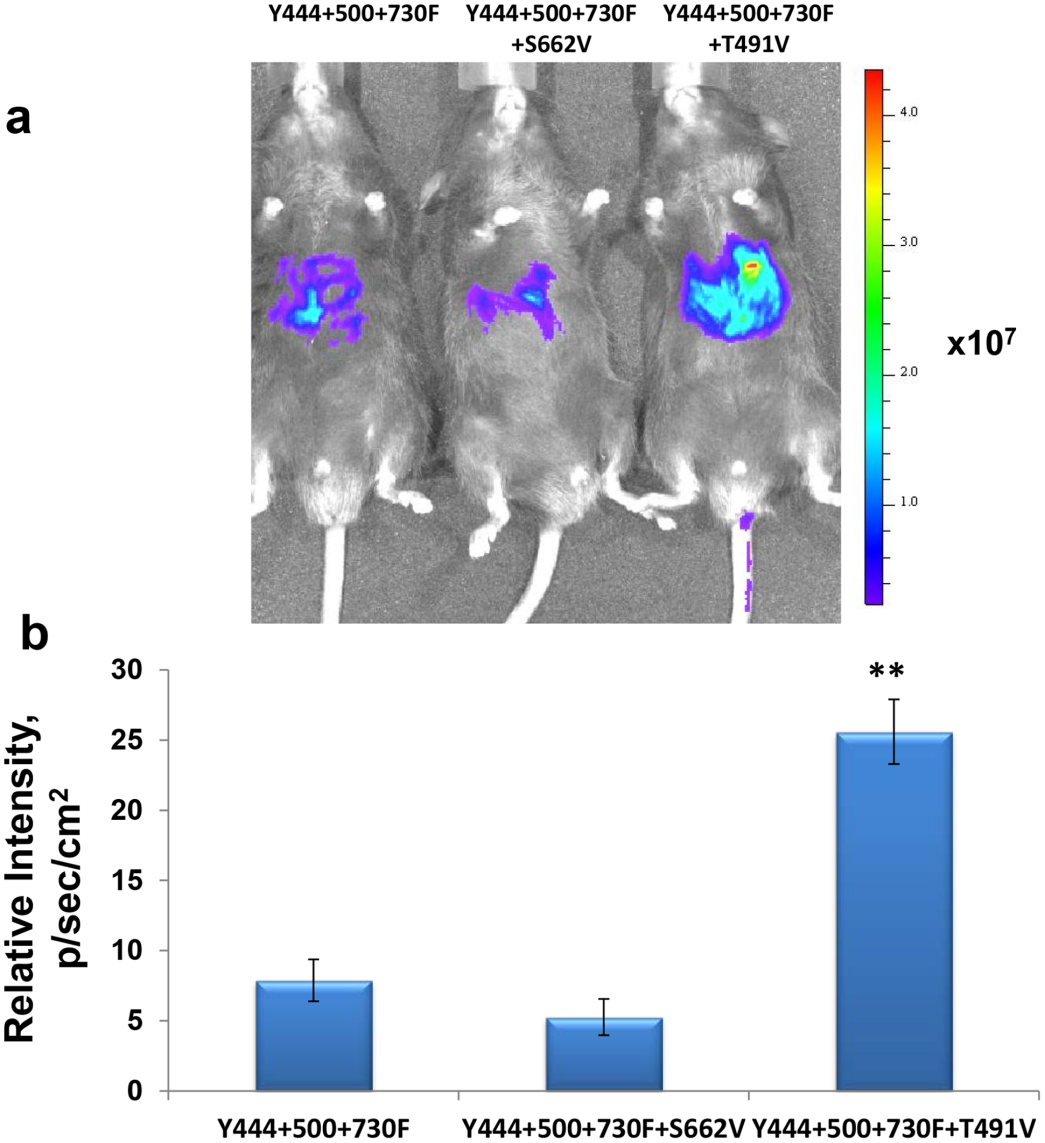
*In vivo* imaging of luciferase gene expression following tail vein injection of multiple site-directed AAV2 capsid mutants. C57BL/6 mice were injected with 1×10e10 vg/animal of several most efficient mutant scAAV vectors carrying luciferase gene. Live images were taken to analyses difference in luciferase activity. The visual output represents the number of photons emitted/second/cm^2^ as a false color image where the maximum is red and the minimum is blue (**a**) and relative signal intensity (**b**). *P<0.005 was considered as significant.

Finally, we evaluated the transduction efficiency of the optimized AAV2 vectors in a murine model *in vivo*. Each of multiple-mutant vectors was packaged with a single-stranded firefly luciferase (Fluc) AAV2 genome, and ∼1×10^10^ vgs of each vectors were injected intravenously into C57BL/6 mice (n = 3 for each group). Levels of expression of Fluc gene, assessed two weeks post-injection by bioluminescence imaging, showed that expression from the Y444+500+730F+T491V quadruple-mutant vector was ∼3-fold higher than that from the tyrosine triple-mutant vector. One representative animal from each group and the quantification of these data are presented in **Fig. 6a and b**. Consistent with the data obtained *in vitro,* the addition of S662V mutation had a negative effect on the transduction efficiency of both the tyrosine-triple-mutant and the tyrosine-threonine quadruple-mutant vectors.

## Discussion

Recombinant AAV-based vectors are attractive delivery vehicles for gene replacement therapy as a potential treatment for a variety of genetic disorders. Although AAV vectors have been used successfully in many animal models, and recently shown efficacy in several clinical trials, a number of steps in the life cycle of AAV continue to appear to limit the effectiveness of these vectors in gene therapy. Some of these steps include intracellular trafficking, nuclear transport, uncoating, and viral second-strand DNA synthesis [30], [31], [32]. Gaining a better understanding of the fundamental aspects of the AAV-host cell interaction, has, and will continue to, lead to improvements in the efficiency of AAV-mediated gene delivery and transgene expression.

The simple organization and natural plasticity of AAV structural and regulatory components provide a unique opportunity to manipulate the viral capsid and the genome to develop customized recombinant vectors with distinctive features. Significant progress has been made in the past decade to improve the specificity and the transduction efficiency of recombinant AAV vectors. For example, specific mutations in the viral inverted terminal repeat (ITR) sequences have led to development of self-complementary AAV (scAAV) vectors, which overcome the rate-limiting step of viral second-strand DNA synthesis, and dramatically increase transgene expression levels in various types of the cells and tissues [33], [34]. Additional studies on capsid structure analyses, combined with a wealth of information emanating from mutagenesis studies on the capsid genes, have led to the identification of specific regions which play a critical role in vector encapsidation, tissue-tropism, and intracellular trafficking of these vectors [35], [36], [37], [38], [39], [40].

In our recent studies, we documented that substitution of surface-exposed specific tyrosine (Y) and serine (S) residues on AAV2 capsids significantly increased the transduction efficiency of these vectors, both *in vitro* and *in vivo*, presumably by preventing phosphorylation, subsequent ubiquitination, and proteasome-mediated degradation [12], [13], [14], [24]. Since surface-exposed specific threonine (T) residues on AAV2 capsids would likewise be expected to undergo phosphorylation, in the present study we systematically mutagenized each of the 17 surface-exposed T residues, and identified several single-mutant vectors that could increase the transduction efficiency up to 4-fold. Combinations of multiple T mutations on a single capsid identified modifications which further augmented the transduction efficiency up to ∼10-fold, compared with that of the WT AAV2 vector in HEK293 cells. It is of interest to note that two independent groups have previously reported mutations of specific T residues on AAV2 capsids. For example, Lochrie *et al*. [35] targeted the T residues at positions 330, 454, 455, 491, 503, and 550 in a tour de force effort to identify surface regions which bind antibodies, and DiPrimio *et al*. [41] targeted the T residue at position 659 in an effort to identify regions critical for capsid assembly and genome packaging. In both studies, the T residues were substituted with either alanine (A), serine (S), or lysine (K) residues, or by peptide substitution. However, no increase in the transduction efficiency of any of the mutant vectors was observed. In contrast, in our studies, we substituted the surface-exposed T residues with valine residues. This further corroborates our recent observation of the critical role played by specific amino acid type in modulating the biological activity of AAV vectors [12], .

When the most efficient threonine-mutation (T491V) was combined with a previously reported tyrosine triple-mutation (Y444+500+730F) [14] to generate a Y-T quadruple-mutant (Y444+500+730F+T491V) vector, the transduction efficiency of this vector was ∼2–3-fold higher than the tyrosine triple-mutant vector in murine hepatocytes, both *in vitro* and *in vivo*. However, combining the most efficient S-mutation (S662V) [12] with the tyrosine triple-mutation negatively affected the transduction efficiency of the Y-S quadruple mutant (Y444+500+730F+S662V) vector as well as the Y-S-T pentuple-mutant (Y444+500+730F+S662V+T491V) vector. Although several other combinations showed greater transduction efficiency compared with the WT AAV2 vector, neither combination of similar (quadruple, pentuple or sextuple-tyrosine; and triple and quadruple-threonine mutants), nor combination of the best performing YST mutations reached the level of expression from the triple-tyrosine mutant vector (Table S1). In view of the large number of combinations of mutations tested in the current studies, we focused on the mutations that significantly increased the transduction efficiency over that from our most efficient previously published triple-tyrosine mutant vector. However, it is possible that additional superior combinations could be identified with even more mutations, and our explanation that phosphorylation of specific amino acids in AAV capsid alone accounts for the observed differences may be inadequate.

**Figure 7:**
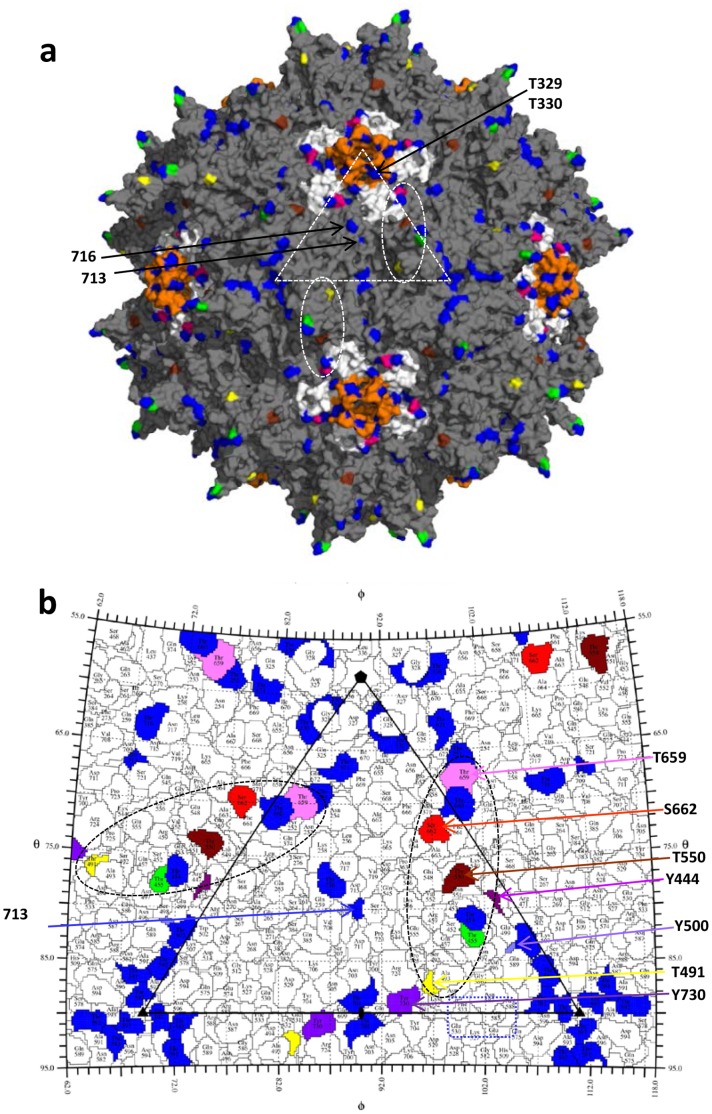
The AAV2 capsid surface. (**a**) The capsid surface of AAV2 (grey) with the 17 surface threonine residues mutated in blue (251, 329, 330, 454, 503, 581, 592, 597, 660, 671, 701, 713, 716), green (455), yellow (491), brown (550), and pink (659). The surface location of T329, T330, T713 and T716 are indicated by arrows. The five-fold symmetry related DE loops (between the βD and βE strands) are colored in orange. The HI loops (between the βH and βI strands) are colored white and S662 located in this loop is in red. The white dashed triangle in (a) depicts a viral asymmetric unit bounded by a five-fold axis and two three-fold axes with a two-fold axis between the three-folds. Dashed ovals delineate the approximate footprints (2/60) of threonine residues that affect transduction when mutated. (**b**) A “Roadmap” projection [23] of the AAV2 capsid surface residues within a viral asymmetric unit. The areas covered by AAV2 surface threonines and S662 are colored as in (a). The residues in the tyrosine triple mutant residues, 444, 500, and 730 are shown in shades of purple. Dashed ovals are as described in (a). Dashed rectangle (blue) shows residues previously determined to be important in heparin sulfate receptor binding for AAV2 and AAV6 [37], [50].

The 17 AAV2 surface-exposed threonine residues are scattered throughout the capsid. Four of the mutations, T329V, T330V, T713V, and T716V, resulted in significant defects in assembly and vector production and could not be further characterized. Residues 329 and 330 are located in the a surface loop (DE loop) located between the βD and βE strands of the core β-barrel of the AAV2 VP3 structure [20]. Five of these loops, from icosahedral five-fold symmetry related VP3s assembly a channel at this axis which connects the interior and exterior surfaces of the capsid (Fig. 7a). As was observed in a previous study by Bleker *et al*. [43], titers for these mutants were significantly reduced consistent with a role for the channel in genome packaging. Residues 713 and 716 are located on the wall/raised capsid region between the depressions at and surrounding the icosahedral two- and five-fold axes, respectively (Fig. 7a and b). Their side-chains participate in polar interactions with symmetry related VP3 monomers and it is likely that mutation results in a defect in capsid assembly. A role in capsid assembly for residues located at the icosahedral two-fold axis is consistent with a recent report by Naumer *et al*. in which they observe that the AAV2 residues which mediate the interaction with the assembly-activating protein (AAP) are located at this capsid region [44].

Residues T455, T491, T550, and T659, showing an increased transduction phenotype when mutated to valine or alanine, are located on the protrusions which surround the icosahedral three-fold axis (T455, T491, and T550) or on the HI loop (between βH and βI of the core β-barrel) (T659) which is lies on the depression surrounding the channel at the icosahedral five-fold axis of the AAV2 capsid (**Fig. 7**). The residues on the protrusion, a prominent feature on the capsid assembled from two VP3 monomers, are located close to the top (455), side facing the two-fold depression (491), and side facing the depression surrounding the five-fold (550), respectively, of the protrusions. This AAV region contains the most variability in sequence and structure, and with the exception of residue 659, the other three threonines residues are located to define VP3 variable regions (VRs) [45]. Along with T659, these residues form a footprint on the capsid surface that extends over the top of the protrusion towards the depression surrounding the icosahedral five-fold axis (Fig. 7a and b). Their surface exposure is consistent with the potential to interact with host molecules, which could include kinases. Interestingly, this footprint is flanked by the residues in the triple-tyrosine mutant, Y444, Y500, and Y730, with T491 located proximal to tyrosine residue Y730 in a depiction of the capsid surface amino acids (Fig. 7b). This residue, which sits in the depression at the icosahedral axis of the capsid, showed the highest increase in transduction compared to WT AAV2 when of the seven surface-exposed tyrosines where mutated to phenylanine residues [13]. Significantly, the two-fold capsid region is observed to undergo pH-mediated structural transitions when the homologous AAV8 was examined at the conditions encountered during trafficking in the endocytic pathway [46]. Thus while the exact underlying molecular basis for the observed improvement in transduction for the T491V, and quadruple mutant (Y444+500+730F+T491V) are not immediately apparent, the proximal location of T491 and Y730 supports a role for the icosahedral two-fold axis in AAV cellular trafficking, including modifications that likely target the capsid for degradation in the proteasome. These results highlight a need for additional studies to delineate the role each of the critical Y, S, and T residues in various steps in the life cycle of AAV vectors. For example, it is possible that the mutations of the AAV2 could be improving transduction efficiency through altered receptor binding mechanisms. Residues mediating AAV2 and AAV6 interaction with heparan sulfate receptors, R585 and R588, and K531 (structurally equivalent to E530 in AAV2), respectively, are close to this foot (Fig. 7b), and residues 491 and 500, in VRV, are located in one of two large regions on the surface of the AAV2 capsid that has been implicated in binding to the LamR receptor in AAV8 [47]. Amino acids in VRV also play a role in the AAV9 capsid binding to its glycan receptor, galactose.

The decreased transduction efficiency phenotype of the mutants containing the S662V mutations is difficult to explain given the location of this residue within the footprint delineated by the residues which enhance transduction when mutated to eliminate potential phosphorylation (Fig. 7a and b). In addition, it was previously shown that a mutation of this residue to valine improved transduction relative to WT AAV2 [12]. Residue S662, like T659, is located in the HI loop which extends over adjacent five-fold symmetry related VP3 monomers and likely plays a role in stabilizing the pentameric subunits. However the serine side-chain is not engaged in any inter- or intra-subunit interactions, and while the HI loop has been reported to be a determinant of capsid assembly and genome packaging [41], it tolerated single amino acid substitution [12]. Thus its effect is likely due to the abrogation of a capsid interaction utilizing the footprint containing the triple-tyrosine mutant residues and T491. Significantly, the phenotypes for mutations in nearby amino acids that make up the HI loop, for example, amino acid residue 664, substituting either serine (mut45subSer14) or a FLAG epitope (mut45SubFLAG10), were non-infectious or not assembled into viral capsid [48]. However, an HA insertion at the same position produced capsids that were partially defective, yet still bound heparin [48]. These observations, once again, highlight the fact that the nature of the amino acid substitution, in additional to the region of the capsid, is an important consideration while engineering optimized gene therapy vectors.

Although, we were unable to experimentally document whether any of the surface-exposed T residues which when mutated improve transduction was phosphorylated by a putative cellular kinase, based on our previous studies with Y-mutant AAV2 vectors [25], [49], it is reasonable to suggest that phosphorylation of these residues could negatively impact intracellular trafficking and nuclear transport of AAV2 vectors. We base this contention on our current studies in which we documented that whereas only ∼45% of the vector genomes delivered by the WT AAV2 vectors were present in the nucleus at 48 h post infection, >90% of the vector genomes delivered by the Y-T quadruple-mutant vector were present at the same time point. This indicates improved trafficking kinetics for the mutant which would be consistent with reduced re-direction to the proteasome. The modest (∼2–3-fold) increase in the transduction efficiency of these vectors compared to the tyrosine triple-mutant vectors is also consistent with the ∼10% increase in nuclear vector genome delivery, i.e. ∼90% compared to ∼80%.

The current studies raise the question of whether further optimization of AAV2 vectors by targeting surface-exposed amino acid residues involved in capsid phosphorylation is feasible. The various combinations of surface tyrosine, serine, and threonine modifications clearly showed that there is an optimal combination to achieve maximal augmentation. These studies also highlighted the requirement for specific residue types in AAV interactions during infection and for enhancing transduction. It is possible that the individual mutations, which did not show a significant increase in the transduction efficiency as single changes, can form superior vectors when combined in a single capsid. However, considering the large number of possible mutation combinations that would have to be produced and evaluated, it is not possible to identify such combinations empirically. Also, the transduction efficiency of novel capsid-modified vectors can be variable for different cell types, and depends on the expression profile and the levels of activity of the kinases involved in AAV capsid phosphorylation [12]. Another possibility for further capsid improvement is to target tyrosine, serine, and threonine residues which are not surface-exposed on the capsid but can be accessible for phosphorylation by kinases during various steps of intracellular trafficking of the virus since the capsid is expected to undergo conformational changes during this process. These possibilities require additional studies.

The studies have resulted in the development of a novel optimized quadruple-mutant (Y444+500+730F+T491V) AAV2 vector which is capable of mediating high-efficiency transduction of hepatocytes. This mutant holds promise as a potential vector for liver-directed gene therapy. Furthermore, most of the threonine residues mutated are conserved in other clinically relevant AAV serotypes, thus their modification would significantly add to the current repertoire of optimized AAV vectors for potential use in human gene therapy.

## Supporting Information

Table S1 **Mutations of surface-exposed tyrosine (Y), serine (S), and threonine (T) residues on the AAV2 capsid.**
(DOCX)
