# Supplementary material for: Optimization of the Capsid of Recombinant Adeno-Associated Virus 2 (AAV2) Vectors: The Final Threshold?
Source: PLoS One. 2013 Mar 19;8(3):e59142. doi: 10.1371/journal.pone.0059142 (PMC3602601; doi:10.1371/journal.pone.0059142)
Supplement: Table S1 — Mutations of surface-exposed tyrosine (Y), serine (S), and threonine (T) residues on the AAV2 capsid. (DOCX) [file pone.0059142.s001.docx]

**Table S1:** Mutations of surface-exposed tyrosine (Y), serine (S), and threonine (T) residues on the AAV2 capsid.

| **Single Mutations** | **Double Mutations** | **Triple Mutations** | **Multiple Mutations** |
| --- | --- | --- | --- |
| Y252F | Y252+730F | Y444+500+730F | Y272+444+500+730F |
| Y272F | Y272+730F | Y730F+S662V+T491V | Y272+444+500+730F |
| Y444F | Y444+730F | S458+492+662V | Y272+444+500+730F |
| Y500F | Y500+730F | T455+550+491V | Y272+444+500+700+730F |
| Y700F | Y700+730F | T550+659+491V | Y272+444+500+704+730F |
| Y704F | Y704+730F |  | Y252+272+444+500+704+730F |
| Y730F | Y444+550F |  | Y272+444+500+700+704+730F |
| S261V | S458+492V |  | Y252+272+444+500+700+704+730F |
| S264V | S458+662V |  | Y444+500+730F+T491V |
| S267V | S492+662V |  | Y444+500+730F+S458V |
| S276V | T455+491V |  | Y444+500+730F+S662V+T491V |
| S384V | T550+491V |  | Y444+500+730F+T550+T491V |
| S458V | T659+491V |  | Y444+500+730F+T659+T491V |
| S468V | T671+491V |  |  |
| S492V | Y730F+T491V |  |  |
| S498V | S662V+T491V |  |  |
| S578V | Y730F+S662V |  |  |
| S658V |  |  |  |
| S662V |  |  |  |
| S662A |  |  |  |
| S662D |  |  |  |
| S662F |  |  |  |
| S662H |  |  |  |
| S662N |  |  |  |
| S662L |  |  |  |
| S662I |  |  |  |
| S668V |  |  |  |
| S707V |  |  |  |
| S721V |  |  |  |
| T251V |  |  |  |
| T329V |  |  |  |
| T330V |  |  |  |
| T454V |  |  |  |
| T455V |  |  |  |
| T491V |  |  |  |
| T503V |  |  |  |
| T550V |  |  |  |
| T597V |  |  |  |
| T592V |  |  |  |
| T581V |  |  |  |
| T671V |  |  |  |
| T659V |  |  |  |
| T660V |  |  |  |
| T701V |  |  |  |
| T713V |  |  |  |
| T716V |  |  |  |

The first letter corresponds to the amino acid in the wild-type AAV2 capsid, the number is the VP3 amino acid position that was mutated, and the last letter is the mutant amino acid.
